# Middle ear glandular neoplasms: adenoma, carcinoma or adenoma with neuroendocrine differentiation: a case series

**DOI:** 10.1186/1757-1626-0002-0000006508

**Published:** 2009-03-13

**Authors:** Issam Saliba, Anne-Sophie Evrard

**Affiliations:** 1 Department of Otorhinolaryngology, Head & Neck surgery, Montreal University Hospital Center (CHUM), Hôtel-Dieu Hospital, Montreal, Quebec, Canada

## Abstract

**Introduction:**

Middle ear glandular neoplasms are infrequent causes of a middle ear mass. They can have exocrine and/or neuroendocrine differentiation. It is currently thought that these tumors are indistinguishable each from another. Herein, we present a new case of a middle ear glandular neoplasm. Our objective is to review all cases described in the literature in order to identify the clinical features, the gross pathology, the histopathology, the immunohistochemistry, to discuss the differential diagnosis, the treatment, the rate of recurrence, the follow-up, the incidence of metastasis, the prognosis and to present a new classification of middle ear glandular neoplasm.

**Case presentation:**

**Methods**

We performed a MEDLINE database search for MEA-related articles published between 1950 and March 2008. The information from the reports was analyzed.

**Results**

Ninety-four patients with a middle ear adenoma are included in this report. We uncovered 75 patients with a carcinoid tumor and 19 patients with a middle ear adenoma diagnosis; the most common presenting symptom was a conductive hearing loss. Middle ear adenomas are lesions that are typically white, gray or reddish brown. They are grossly vascular and well circumscribed, but not encapsulated, and can entrap and destroy the ossicles. Histologically, the cuboidal to low columnar cells are arranged in a solid, trabecular or glandular architecture. The tumor's cells are immunohistochemically positive for a variety of keratin antibodies and most of them are also positive for neuroendocrine markers. Surgical excision is the treatment of choice. Local recurrence following complete excision is quite uncommon and metastases are rare.

**Conclusions**

Our study and the review of the literature showed adenomas and carcinoid tumors of the middle ear to be essentially indistinguishable benign tumors with metastatic potential. Based on the presence or absence of immunohistochemical markers and metastasis, we have classified these lesions into three types. Complete surgical treatment is recommended with an indefinite follow-up for possible recurrence.

## Introduction

Middle ear glandular neoplasms are seldomly the source of a middle ear mass. Hyams [1] was the first to describe a series of these tumors in 1976. He designated these tumors as middle ear adenomas (MEA). In 1980, Murphy et al [2] described a similar, if not identical, tumor and designated it a carcinoid tumor because of the ultrastructural evidence of a neuroendocrine differentiation.

Middle ear glandular neoplasms can present both a neuroendocrine and an epithelial differentiation. This has led some early authors to maintain that neuroendocrine (carcinoid) tumors and middle ear adenomas were different tumors [3]. However, most authorities currently think that there is a single primary low-grade glandular neoplasm of the middle ear. They are describing carcinoid tumors of the middle ear identical to the tumors reported as middle ear adenomas. This has led most authorities to conclude that a middle ear adenoma, or the variant neuroendocrine adenoma, was a preferred nomenclature as opposed to that of a carcinoid tumor since these terms imply a benign behavior, which is consistent with the vast majority of cases.

In this paper, we present a new case of a middle ear adenoma / carcinoid tumor. Our objective is to review all cases described in the literature in order to identify the clinical features, the gross pathology, the histopathology, the immunohistochemistry, to discuss the differential diagnosis, the treatment, the rate of recurrence, the follow-up, the incidence of metastasis, the prognosis and to present a new classification of middle ear glandular neoplasm.

## Materials and Methods

We performed a MEDLINE database search for MEA-related articles published between 1950 and March 2008. The electronic search was conducted with the keywords "carcinoid", "middle ear adenoma", "middle ear mass", "middle ear tumor", "ear adenoma", "tympanic mass", "temporal bone tumor", "neuroendocrine", and "neuroendocrine immunohistochemistry". The results yielded 74 relevant studies, in which 93 patient records were transcribed. In addition, we adjoin our case to this series. The information from the reports was analyzed to characterize the clinical aspects, the radiologic findings, the histopathology and immunohistochemistry, the treatment, the rate of recurrence, the follow-up, and the incidence of metastasis related this disease.

## Case Presentation

In January of 2006, a 32-year old man presented at our otolaryngology department for a second opinion regarding a seven-year history of right aural fullness and mild hearing loss. Five years prior to this consult, he had been examined by an otorhinolaryngologist for a right-sided otalgia associated with a right facial paralysis. He had been given oral amoxicillin and steroids for a probable middle ear otitis complicated with a facial paralysis. A few months after this episode, an antero-inferior myringotomy was performed for a presumed middle ear effusion and revealed neither liquid, nor mass. He had never noticed any discharge, vertigo, tinnitus, visual symptoms or headaches, as well as any other significant past or present disease.

**Figure 1:**
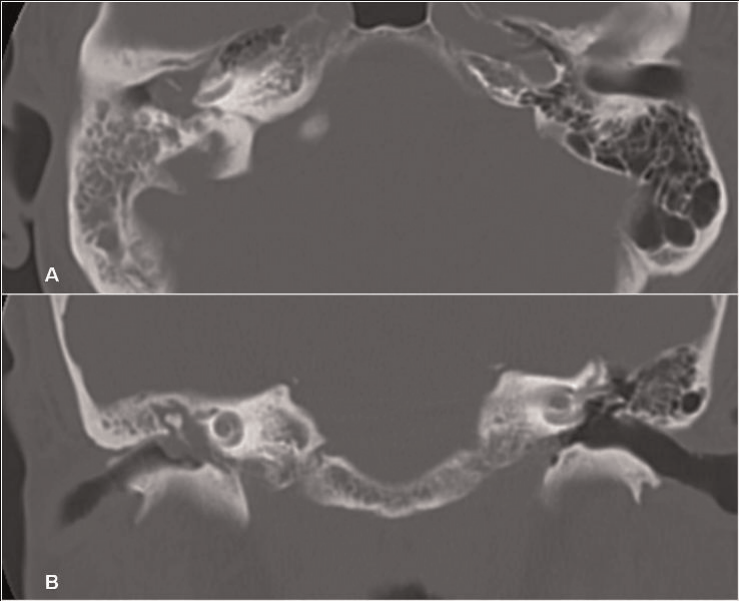
**Axial (A) and coronal (B) views of a mastoid computerized tomography (CT) scan showing a right mastoid and middle ear opacity**. The tympanic membrane is pressed laterally by the mass.

The otoscopy showed the presence of a posterosuperior retrotympanic mass with normal tympanic membrane. The hearing test revealed a right mixed hearing loss. A computed tomography (CT) scan of the temporal bone revealed opacity in the superior part of the middle ear extending into the mastoid cavity (Figure 1). There was neither bone erosion, nor ossicular destruction and it was compatible with a chronic otomastoiditis.

In March 2006, he underwent a right postauricular canal wall-up mastoidectomy and an extended facial recess approach procedure. A multilobulated, polypoid, fibrotic, grey tumor was observed filling the middle ear cavity. The mass was located in the mesotympanum, epitympanum and hypotympanum components. There was a prolongation into the antrum, the mastoid, and the Eustachian tube without any involvement of the facial nerve or evidence of bone erosion. Even if the ossicles were embedded in the tumor, there was no ossicular chain erosion, but the malleus was fractured, the incus and the stapes were dislocated. The malleus and incus were removed and a piece of fascia was placed on the oval window to avoid a perilymphatic fistula. The intraoperative frozen study was not conclusive. A total macroscopic excision of the lesion was performed; a second look procedure for ossicular chain reconstruction was to be performed five months later. There were no major postoperative complications. The hearing test showed a right severe sensorineural hearing loss, which was improved by a hearing aid.

**Figure 2:**
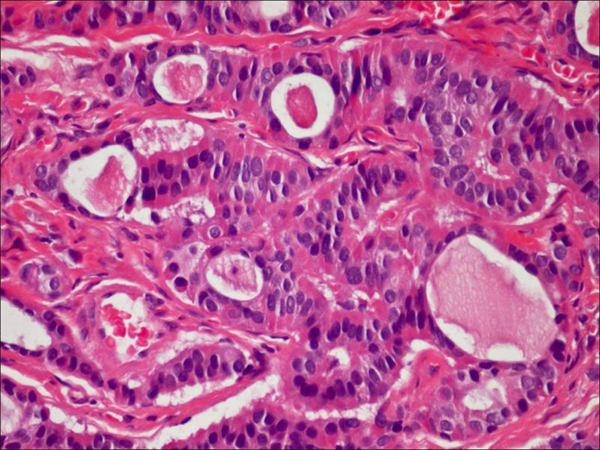
**Middle ear adenoma**. Tumor composed of small glands lined by a single layer of uniform cuboidal cells with an intraluminal eosinophilic secretion. No mitotic activity or necrosis (original magnification 400X).

**Figure 3:**
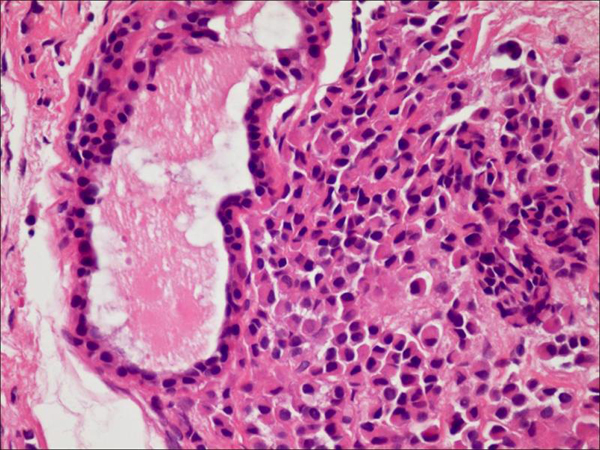
**Middle ear adenoma**. Minor foci of tumor composed of sheets of loosely cohesive cells with moderate to abundant eosinophilic cytoplasm and eccentrically placed nuclei (plasmacytoid morphology) (original magnification 200X).

**Figure 4:**
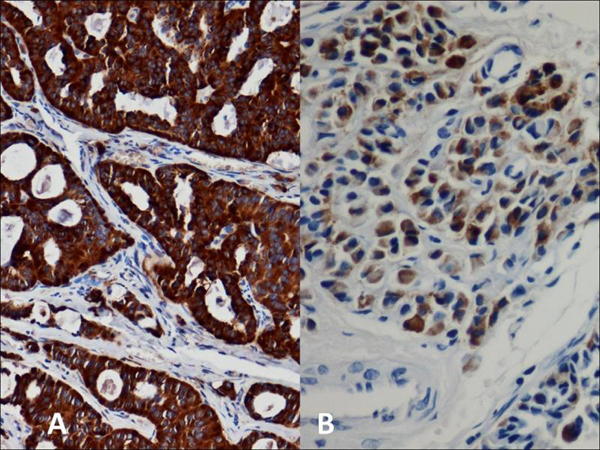
**Middle ear adenoma**. Immunohistochemical stains for synaptophysin **(A)** and chromogranin **(B)** show strong positivity within tumor cells.

**Figure 5:**
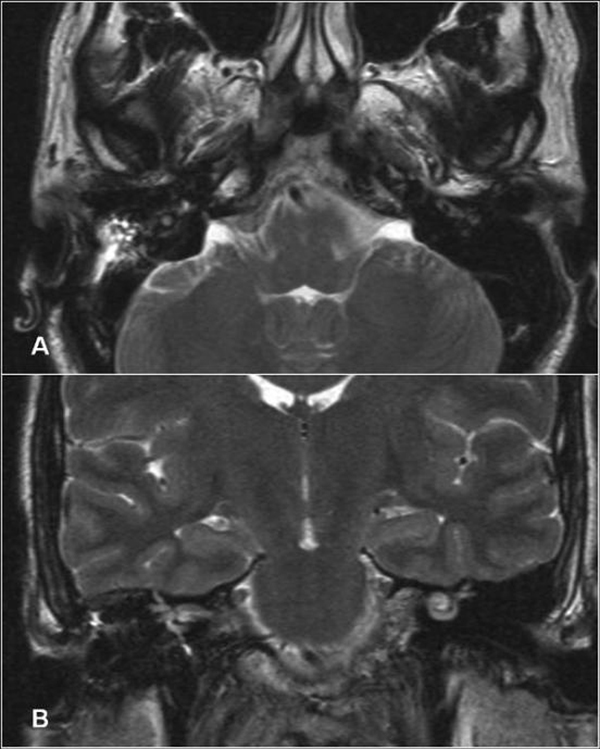
**Axial (A) and coronal (B) T2-weighted magnetic resonance imaging (MRI) scan one-year post-operative excision showing no sign of recurrence**.

Histologic examination of the excised tumor showed an epithelial neoplasm with a predominantly glandular architecture embedded in fibrous tissue. The tumor was composed of cuboidal cells with uniform nuclei and no identifiable mitotic activity or necrosis (Figure 2). Focally, the tumor cells had a well-developed plasmacytoid morphology (Figure 3). Single cells were also seen infiltrating into the fibrous stroma. An immunohistochemical evaluation showed strong positivity for neuron specific enolase (NSE) and synaptophysin and weak positivity for chromogranin (Figure 4). The tumor was suspected of having the typical morphology and immunophenotype of a middle ear adenoma/carcinoid. The patient was clinically monitored for two years through the T1 and T2-weighted magnetic resonance imaging (MRI) images of the temporal bones with the administration of a paramagnetic contrast material. He is currently disease free (Figure 5).

## Results

We uncovered 74 relevant studies. Ninety-four cases of middle ear adenoma/carcinoid tumor of the middle ear have been published; including our own afore-described case. We identified 75 patients with a diagnosis of middle ear carcinoid tumor and 19 patients with a diagnosis of middle ear adenoma. The patient's age in this series ranged from 16 to 77 years with a mean age of 44.6 years. The incidence of right versus left involvement was equal. There were 41 women and 53 men yielding a female to male ratio of 1:1.3.

### Clinical Presentation

Hearing loss (HL) was the most common presenting symptom (86.3%). The majority had conductive HL (Table I); four patients had sensorineural hearing loss (SNHL), one from a previous surgery. Nine patients had a facial function weakness; seven had resolution after treatment while two of them had direct invasion requiring sacrificing the nerve. The duration of the symptoms varies from seven days to twenty years, with an average of 38 months.

**Table 1:** Clinincal, radiographic, intraoperative findings and follow up of middle ear adenoma (19 cases) and carcinoid tumor (75 cases) reported in the literature including our case

Clinical presentation	Radiographic presentation			Intraoperative findings Tumor localisation / Middle ear with extension to:	Follow up
Hearing loss 86.3%	CT	Mastoid	18%	Mesotympanum 65%	Mean : 53 months [2-396]
Aural fullness 33%	scan	EAC	3%	Hypotympanum 53%	
Tinnitus 27.6%	(68%)	Eustachian tube	2%	Epitympanum 49%	Recurrence rate: 12.7%
Otalgia 15%		Fallopian canal	2 %	Aditus ad antrum 15%	Time for recurrence: 108 months
Otorhhea 11.4%		Jugular foramen	2 %	Mastoid 15%	[13-516]
Facial weakness 11%		Tegmen antrilysis	< 1 %	Eustachian tube 7%	Local recurrence (N=8): 67%
Mean duraion of	MRI	Low to moderate signal	T1	No precise description 20%	Local + regional recurrence (N=4): 33%
symptoms: 38	(13%)	High signal	T2		Disease free from last treatment:
months		Enhancement	Contrast		26 months [5-60]

### Radiographic Studies

A CT scan of the temporal bones was performed in 68% of patients and demonstrated a soft tissue mass in the middle ear. The ossicles were embedded by the tumor without bone or ossicular erosion in all the cases except for one patient (Table I). An evaluation including a CT of the neck, chest, and abdomen was done in two patients and did not reveal a metastatic disease. A few cases (11%) have had plain mastoid x-rays or conventional tomography (4%) and revealed, in most of them, opacity of the mastoid air cells. In 5% of cases, no radiological investigation was completed. In three cases, a postoperative CT scan of the temporal bones was completed, the delays were not specified. In our case presented here, an MRI of the temporal bone was completed on the postoperative first and second year.

### Intraoperative Findings

Grossly, the tumors present different appearances. The aspect was clearly described in 48 patients (Table I). It was gray-white in 35%, fibrotic, polypoid or multilobulated in 15%, cholesterol-like or fatty in 9%, yellowish jelly-like in 25%, reddish non vascular and non pulsatile in 14%, cholesteatoma-like in 2%, and of unspecified color for the remaining patients. The epicenter of the tumor was located in the middle ear with an extension to the mesotympanum in 65% of cases, to the hypotympanum in 53%, and to the epitympanum in 49%. The aditus ad antrum was involved in seven patients, the mastoid in ten patients, and the Eustachian tube in four patients. In 38 patients, there was no precise description of the tumor's location. The tumors may be relatively well-circumscribed, but not encapsulated; they peel off the bony walls of the middle ear, but may entrap and destroy the ossicles. Ossicular involvement occurred in 70% of cases, but only eight cases had ossicular erosion. The majority of reports indicate that ossicles were removed with the tumor excision. Facial paresis was identified in nine cases and the nerve was frankly invaded in only two cases.

### Histopathology

In this series, 75 cases were diagnosed as carcinoid tumor because of their positivity to neuroendocrine markers on immunohistochemistry (Table II). The 19 remaining cases were diagnosed as middle ear adenoma based on the gross pathology and histopathology. Neuroendocrine markers were done just in one out of the nineteen cases and results were negative. No molecular genetic studies have been performed on middle ear adenoma.

**Table II:** Immunohistochemical Data of Reported Cases per Years represented by percentage of positive cases

Immunohistochemistry	2008 - 200022 cases	1999 - 199030 cases	1989 - 196723 cases	Total of positive test(2008-1967)
Chromogranin A	86 %	53 %	25 %	44 %
Neuron Specific Enolase (NSE)	59 %	51 %	29 %	39 %
MET secretory granule	4.5 %	33 %	58 %	26 %
Synaptophysin	50 %	24 %	-	20 %
Serotonin	5 %	40 %	25 %	20 %
Vimentin	14 %	27 %	21 %	17 %
Cytokeratin	14 %	30 %	12 %	16 %
Keratin	14 %	20 %	21 %	15 %
Pancreatic polypeptide (PP)	-	30 %	21 %	15 %
Grimelius	5 %	20 %	17 %	12 %
Glucagon	-	17 %	17 %	10 %
Epithelial Membrane Antigen (EMA)	18 %	-	17 %	8 %
Protein S-100	-	10 %	12 %	6 %

Immunohistochemistry positive result less than 5% (are not reported in the table) of cases for the period from 1967 to 2008: Cytokeratin AE1 / AE3, Cytokeratin cocktails, Cytokeratin KL 1, Cytokeratin CAM, Carcino Embryogenic Antigen (CEA), Periodic Acid-Schiff (PAS), Tissue polypeptide antigen (TPA), Calcitonin, Lyzozyme, Leu-7, Cholecystokinin, Fontana-Masson, Protein gene product 9.5 (PGP), Stomatostatine, Vasoactive intestinal polypeptide (VIP).

### Treatment

All patients were primarily treated with surgery. Transcanal tympanotomy (TM) or tympano-mastoidectomy approaches were used. Seventeen patients underwent an initial biopsy followed by a more definitive procedure such as canal wall up (CWU) mastoidectomy, canal wall down (CWD) mastoidectomy, or radical mastoidectomy (RM). A secondary ossicular reconstructive procedure was only documented in our reported case. The definitive surgical treatment was through a transcanal TM in 46% of patients, CWU mastoidectomy in 21% of patients, CWD mastoidectomy in 16%, and RM in 22% of patients. Four patients underwent a subtotal petrosectomy, three patients simply underwent a biopsy, one was treated by the excision of the EAC mass, and eight had unspecified surgery combined with postoperative radiation therapy; a dose ranging from 45 to 60 Gray. Chemotherapy was not prescribed in any other cases.

### Recurrence and Metastasis

Sixty-one patients were disease free at their last follow-up, 21 patients had an unspecified follow-up while twelve (12.7%) patients developed a localized recurrence of the disease (Table I). Only one case out of these twelve patients had MEA. No immunohistochemistry test was done. The average interval from initial treatment to the first recurrence was nine years from a range of 13 months to 43 years. In all recurrences, the initial excision was conservative, leaving the ossicular chain intact. Tympanotomy was associated with a local recurrence rate of 14%, whereas radical mastoidectomy was associated with a local recurrence rate of 9%.

### Follow-up

The disease-free interval after definitive surgery was available for 73 of the patients. This interval ranged from two months to 33 years with an average of 53 months. The follow-up for the patients treated with radiotherapy was not indicated. The interval between initial surgery and first recurrence was available for eleven patients and ranged from 16 to 396 months with an average of 52 months. The duration of the follow-up after treatment for a recurrent or a metastatic disease was available for nine of the patients, ranging from five months to five years with an average of 26 months.

## Discussion

Middle ear adenomas are unusual neoplasms with epithelial and neuroendocrine differentiations. They are composed of two types of cells; exocrine and neuroendocrine in which neuroendocrine granules and sometimes neuropeptides (chromogranin, synaptophysin, serotonin, and pancreatic polypeptide) are detected [4,5].

### Etiology

Carcinoid tumors of the lung are thought to originate from enterochromaffin cells (Kulchitsky cells), which are neuroendocrine normal cells present in the lung. However, epithelial cells with neuroendocrine characteristics are not noted in the middle ear cavity. An undifferentiated pluripotential endodermal stem cell may still be present within the surface mucosa, giving rise to carcinoid neoplasms similar to the Kulchitsky cell [6]. Hyams and Michaels were the first to hypothesize that MEA originated from the mucosal epithelium of the middle ear, but the lack of evidence for surface epithelial derivation leads to the consideration of a stromal precursor [1]; the stroma of the middle ear derived from the mesoderm and the neural crest. The neural crest gives rise to parts of the ossicular chain and the three primary paraganglia. Positive immunohistochemical staining for neuroendocirne tissue, NSE, chromogranin, and/or synaptophysin suggest that adenoma of the temporal bone originates from neuroectoderm. Epithelial and exocrine characteristics are not normal features of these cells and it is plausible that a neuroendocrine neoplasm of the middle ear may originate from a neural crest-derived stem cell.

### Clinical Presentation

The mean age being the forties and there was no gender difference. Patients typically present with conductive hearing loss, aural fullness, and tinnitus. An examination usually reveals a gray-white or fibrotic mass behind an intact tympanic membrane. Facial palsies associated with middle ear carcinoid have been reported in the literature. Krouse et al. presented one patient with transient paresis [7] and similar findings were described in the report of Torske et al., who retrospectively analyzed 48 cases from the archives of the Armed Forces Institute of Pathology [8]. In none of these cases was the nerve infiltrated by the tumor. However, bone dehiscences of the facial canal were described, which could be related to either anatomic abnormalities or the tumor itself. Even in normal ears, the rate of minor dehiscence of the bony canal is rather high. In the report of Nikanne at al [9] and in our case, the facial function returned to normal before any surgical treatment. In these cases "clinically" intact shell around the facial nerve makes every discussion about the damaging mechanism difficult and speculative. Friedmann et al. presented a patient who had a tumor enveloping the facial canal, but without any invasion of the nerve [3]. Ramsey et al reported a case with facial paresis associated with invasion of the facial nerve and an intraneural spread [10]. Knerer et al. reported another patient who required facial nerve sacrifice because of tumor involvement [11]. The carcinoid tumor belongs to the group of APUDomas (Amine Precursor Uptake and Decarboxylation) or endocrine cell tumors. Only Latif and al. reported a carcinoid syndrome from a neuroendocrine tumor of the middle ear [12]. It is the only reported case where the patient complained of diarrhea, abdominal cramps, skin flushing, and bronchoconstiction. Clinical identification is usually misinterpreted as cholesteatoma, chronic otitis media or paraganglioma, schwannoma, hamartoma, squamous cell carcinoma, rhabdomyosarcoma, and papillary adenocarcinoma.

### Radiology

Since the clinical features and otological findings are not characteristic, one must rely on radiological findings. The imaging characteristics of adenomatous middle ear tumors have not been clearly defined because of their rarity. However, the CT of the temporal bone is the key procedure. It highlights a nonspecific opacity, well limited, being able to extend to the whole of the tympanic cavity and the mastoid and posing mainly the differential diagnosis from a glomic tumor. The ossicles are generally embedded in the mass without ossicular or bony erosion. No characteristic differences between the benign and malignant tumors are detectable on a CT [13]. On an MRI, the tumors are isointense or generated a slightly higher signal than white matter on T1-weighted images; there is contrast enhancement. On T2-weighted images, the tumors approximate the signal intensity of gray matter [13]. Although the ossicles are visible as signal voids within the tumor, bone erosion could not be assessed. Indeed, an MRI does not provide preoperative information in addition to that generated by a CT, mainly due to the small size of the tumors. In cases with extension to the posterior cranial fossa or cerebellopontine angle, an MRI is expected to provide additional help in the diagnosis and in planning the resection. CT and MRI in the investigation of a suspected middle ear tumor are recommended.

### Intraoperative Findings

All patients presented with a unilateral disease. Most of the lesions were excised in a piecemeal fashion and they peel off the bony walls of the middle ear, but may entrap and destroy the ossicles. The biological nature of the tumors could not even be assessed intraoperatively. Facial paralysis, bone lyses, or chronic otorrhea can exist in the case of a malignant lesion. None of these symptoms is specific. The surgeon should keep in mind the possibility of a middle ear adenoma especially in the presence of a fibrotic mass behind an intact tympanic membrane.

### Pathological Findings

Microscopically, all tumors were unencapsulated and of moderate cellularity. They were predominantly composed of cuboidal-to-columnar cells with indistinct cytoplasmic borders. The cytoplasm was eosinophilic and homogenous to finely granular. The nuclei tended to be round to oval with minimal pleiomorphism [14]. The chromatin tended to display a "salt-and-pepper" pattern consistent with a neuroendocrine origin. Architectural patterns include glandular, trabecular, solid, and infiltrative. The architecture varied between tumors and within the same tumor.

Immunohistochemistry reveals that the tumor is typically keratin-, vimentin-, pancreatic polypeptide-, and chromogranin-positive, with a lesser number of tumors proving to be neuron-specific-enolase (NSE), synaptophysin-, serotonin- and S-100 protein-positive [15]. Immunohistochemical is also positive with a variety of neuroendocrine-associated markers, including Leu-7, serotonin, and pancreatic polypeptide [16]. We found that chromogranin, NSE, MET, synaptophysin, and serotonin are positive in more than 20% of cases and since 1967, are almost always performed. We suggest that these markers should be tested in all MEA suspected cases to rule out a carcinoid tumor.

### Classification

An analogy between MEA and carcinoid has been proposed. Torske and Thompson have suggested that both are the same tumor [8]. On the other side Ramsey concludes that a carcinoid tumor of the middle ear is the appropriate term and should be considered as a distinct entity from the MEA [10]. Middle ear carcinoid was reported to have a metastatic potential, so it should be considered as a low-grade malignancy [10,17,18]. Some have suggested using neuroendocrine adenoma of the middle ear as a more descriptive term [8,19]. In the nineteen cases of MEA reported here, immunohistochemistry was negative in only one case and it was not performed or unspecified in the remaining cases. Based on these different descriptions and on the presence or absence of markers and metastases, we classified these lesions into three types (Saliba's classification of middle ear glandular neoplasms) (Table III): the most common one (type I) is the neuroendocrine adenoma of the middle ear (NEAME) (76%) (positive immunohistochemistry, negative metastasis) followed by (type II) the middle ear adenoma (MEA) (20%) (negative immunohistochemistry, negative metastasis), and the least common one (4%) (type III) is the carcinoid tumor of the middle ear (CTME) - positive immunohistochemistry, positive metastasis and / or carcinoid syndrome - associated to a metastasis most commonly found in the ipsilateral parotid gland. We propose this classification to discard ambiguity in the diagnosis category, to clarify each group of middle ear glandular neoplasm and for prognostic expectation. Upon this classification, we recognize that only type I tumor can develop metastasis and recurrence could be related only to positive immunohistochemistry cases. In addition, immunohistochemistry was not done in 16 out of 19 MEA reported cases, which suggests that the percentage of MEA probably represents less than 20% of the cases. Pellini et al presented the only case of middle ear free, temporal bone carcinoid metastasis to the cervical lymph nodes [20].

**Table III:** Saliba's classification of middle ear glandular neoplasms based on the presence or absence of markers and metastases

Type	Description	Characteristics	Percentage
**I**	NEAME	Immunohistochemistry (+)Metastasis (−)	76 %
**II**	MEA	Immunohistochemistry (−)Metastasis (−)	20 %
**III**	CTME	Immunohistochemistry (+)Metastasis (+)and / or Carcinoid syndrome (+)	4 %

### Treatment

Complete surgical removal of the neoplasm including the encased ossicles should be the preferred treatment. When the ossicular chain is involved, but not removed, the recurrence of the lesion is more likely to occur. The surgery should be determined on the basis of the clinical and radiological findings. The incidence of recurrence is higher with transcanal tympanotomy (14%) than with a radical mastoidectomy (9%). This is insufficient evidence to suggest superiority of one procedure over another. CWU mastoidectomy with an extended facial recess approach could be an option. In some cases, patients were treated with repeated debulking-excision procedures to preserve the ossicular chain and thus, retain their hearing. Discouraged by surgeons, this technique would require a continued long term clinical follow-up and patient compliance. In order to prevent recurrence, the treatment of choice is total exploration and surgical excision with removal of the ossicular chain [21].

Radiation, chemotherapy and somatostatin analogues have been used in the treatment of gastrointestinal and pulmonary carcinoid tumors, but no data exists for the treatment of middle ear carcinoid. Radiation therapy is not required for these tumors [7]. In fact, secondary malignant transformation is a possible outcome as we have seen with a case of metastatic spread [17].

### Recurrence

In all recurrences, the initial excision was conservative, leaving the ossicular chain intact and the immunohistochemistry, when done, was positive. We noted that all the patients who underwent biopsy followed by definitive surgery had no recurrent disease. Regional metastasis occurred in four patients. They should be managed surgically with a parotidectomy or a neck dissection. Nikanne et al used octreotide scanning in one case in the management of a carcinoid tumor [9]. In another case, a recurrence of middle ear carcinoid was detected by Indium-111(In-111) pentetreotide scintigraphy; its usefulness for the detection of a carcinoid tumor is well known, but has rarely been documented for the detection and follow-up [22].

### Follow-up

The disease-free interval after definitive surgery averaged 53 months. All of the authors recommend a long-term clinical follow-up, but most of them do not specifically recommend a radiological control. When an ossicular chain reconstruction is performed and the usage of cartilage for tympanoplasty middle ear examination becomes impossible, we recommend that either a CT scan or an enhanced MRI be completed.

## Conclusion

Middle ear glandular neoplasms are uncommon well-documented neoplasms of the middle ear. We suggest a prognostic helpful classification based on the presence or absence of markers and metastasis; type I (NEAME), type II (MEA), and type III (CTME). Despite considerable debate over the similarities and differences between these tumors, we believe they are clinically identical, but immunohistochemical results make the difference. This review demonstrates a 12.7% recurrence rate after excision. Surgical management with ossicular chain removal is the recommended treatment to ensure a complete excision. Long-term follow-up is important because of the late recurrences and metastases.

## Consent

"Written informed consent was obtained from the patient for publication of this case report and accompanying images. A copy of the written consent is available for review by the Editor-in-Chief of this journal." The patient prefers to remain anonymous.

## Competing interests

The authors declare that they have no competing interests.

## Authors' Contributions

IS involved in drafting the manuscript, revising it critically for important intellectual content, analysis and interpretation of data and have given final approval of the version to be published. AS have made substantial contributions to conception and design, acquisition of data and have been involved in drafting the manuscript.
